# Application of NMR-based metabolomics for environmental assessment in the Great Lakes using zebra mussel (*Dreissena polymorpha*)

**DOI:** 10.1007/s11306-015-0789-4

**Published:** 2015-02-21

**Authors:** Miki Watanabe, Kathryn A. Meyer, Tyler M. Jackson, Tracey B. Schock, W. Edward Johnson, Daniel W. Bearden

**Affiliations:** 1 Chemical Sciences Division, Hollings Marine Laboratory, National Institute of Standards and Technology, 331 Ft. Johnson Rd., Charleston, SC USA; 2 NOAA Mussel Watch Program, National Oceanic & Atmospheric Administration, National Centers for Coastal Ocean Science, 1305 East West Highway, SSMC4, Room 9202, Silver Spring, MD 20910 USA

**Keywords:** Zebra Mussel, *Dreissena polymorpha*, Mussel Watch, Freshwater, Metabolomics, NMR

## Abstract

Zebra mussel, *Dreissena polymorpha*, in the Great Lakes is being monitored as a bio-indicator organism for environmental health effects by the National Oceanic and Atmospheric Administration’s Mussel Watch program. In order to monitor the environmental effects of industrial pollution on the ecosystem, invasive zebra mussels were collected from four stations—three inner harbor sites (LMMB4, LMMB1, and LMMB) in Milwaukee Estuary, and one reference site (LMMB5) in Lake Michigan, Wisconsin. Nuclear magnetic resonance (NMR)-based metabolomics was used to evaluate the metabolic profiles of the mussels from these four sites. The objective was to observe whether there were differences in metabolite profiles between impacted sites and the reference site; and if there were metabolic profile differences among the impacted sites. Principal component analyses indicated there was no significant difference between two impacted sites: north Milwaukee harbor (LMMB and LMMB4) and the LMMB5 reference site. However, significant metabolic differences were observed between the impacted site on the south Milwaukee harbor (LMMB1) and the LMMB5 reference site, a finding that correlates with preliminary sediment toxicity results. A total of 26 altered metabolites (including two unidentified peaks) were successfully identified in a comparison of zebra mussels from the LMMB1 site and LMMB5 reference site. The application of both uni- and multivariate analysis not only confirmed the variability of altered metabolites but also ensured that these metabolites were identified via unbiased analysis. This study has demonstrated the feasibility of the NMR-based metabolomics approach to assess whole-body metabolomics of zebra mussels to study the physiological impact of toxicant exposure at field sites.

**Electronic supplementary material:**

The online version of this article (doi:10.1007/s11306-015-0789-4) contains supplementary material, which is available to authorized users.

## Introduction

The main focus of conventional toxicology studies has usually been the measurement of toxicant levels in the organism (body burden) and the observation of various correlated physiological end-points such as body weight or survival rate in response to toxicant exposure (Broeg et al. 2005). At the exposure levels that may be present in the natural environment, however, it may be very difficult to detect physiological responses. Because many of the chronic exposure levels in remediated ecosystems may be low, the development of sensitive, high-throughput techniques to detect sub-lethal responses in indigenous organisms is critical for ongoing environmental assessments.

Metabolomics is the systematic study of concentration profiles of endogenous metabolites (the *metabolome*) in biofluids and tissues of a given biological system. This approach provides a novel way to study the changes in the metabolome under various stimuli (Bollard et al. 2005; Lindon et al. 2000; Nicholson et al. 2002). This technique may take a targeted approach (measurement of specific metabolites related to specific prior knowledge or hypotheses) or a non-targeted approach (based on an analytical approach with non-selective detection of compounds for the discovery of interactions in all potential metabolic pathways) that allows the simultaneous detection of a wide range of metabolites (Patti et al. 2012). Many analytical approaches are feasible in metabolomics studies, including mass spectrometry and nuclear magnetic resonance (NMR), each modality having well-known strengths and weaknesses. In exploratory studies, NMR is often used because of its capability for high-throughput analysis, its highly quantitative assessments and its high analytical reproducibility (Naz et al. 2014; Bearden 2014). Metabolomics is applicable to the study of various types of biological samples including whole organism, tissue, cell line and bio-fluid samples (Duarte et al. 2009; Beckonert et al. 2007; Bollard et al. 2005; Viant 2008; Wu et al. 2008). The metabolomics approach has been applied to a wide variety of studies ranging from disease diagnosis, drug toxicity, animal health monitoring, to environmental toxicology (Schock et al. 2010, 2013; Viant 2008; Viant et al. 2003; Nicholson et al. 2002; Kaddurah-Daouk et al. 2008). Previously, metabolomics has been used to study the physiological effect of different toxicant exposure in marine mollusks (Wu et al. 2011; Wu and Wang 2011; Wu et al. 2013; Zhang et al. 2011a; Viant et al. 2003; Jones et al. 2008; Tikunov et al. 2010), and the feasibility of using the metabolomics approach to study organisms from natural habitats has been demonstrated using marine mussels (Hines et al. 2007). These studies have demonstrated that metabolomics is capable of identifying the metabolic responses to various stimuli in organisms in both laboratory exposures and in natural habitats. To date, no studies that we are aware of have been published in the case of freshwater mollusks.

The levels of contaminants in mussels from the Great Lakes have been monitored by the National Oceanic and Atmospheric Administration (NOAA/NCCOS) as a part of the National Status and Trends Mussel Watch Program (MWP) which has been ongoing since 1986 (Kimbrough et al. 2008). In this program, for sampling in the Great Lakes region, non-native freshwater mussels, *Dreissena polymorpha* (zebra mussel) or *Dreissena rostriformis bugensis* (quagga mussel) are collected at multiple sites in Areas of Concern (AOCs) to monitor as a bio-indicator for environmental health effects. The levels of the toxicants in mussel tissue, including metals (e.g. mercury, arsenic, lead and cadmium) and organic compounds [e.g. polychlorinated biphenyls (PCB), and dichlorodiphenyltrichloroethane (DTT)], have been closely monitored over the years; however, the physiological effects on animals due to the assumed toxicant exposure remain unclear.

Herein, we report on the application of NMR-based metabolomics to the analysis of the whole-body metabolome of zebra mussels collected from the Milwaukee Estuary AOC in Lake Michigan (Cooksey et al. 2013) in September 2012. This is an initial feasibility study to determine whether metabolomics could augment standard practices for evaluating ecosystem impairment. The goals of this exploratory study are (1) to evaluate the effects of field protocols and spatial variability in metabolic profiles of zebra mussels; (2) to determine if there is significant correlation between the metabolic profile of zebra mussel tissue and sediment toxicity profiles; and (3) to identify metabolic differences in zebra mussel tissue between the selected reference site and the impacted sites.

## Methods

### Sample collection

**Fig. 1:**
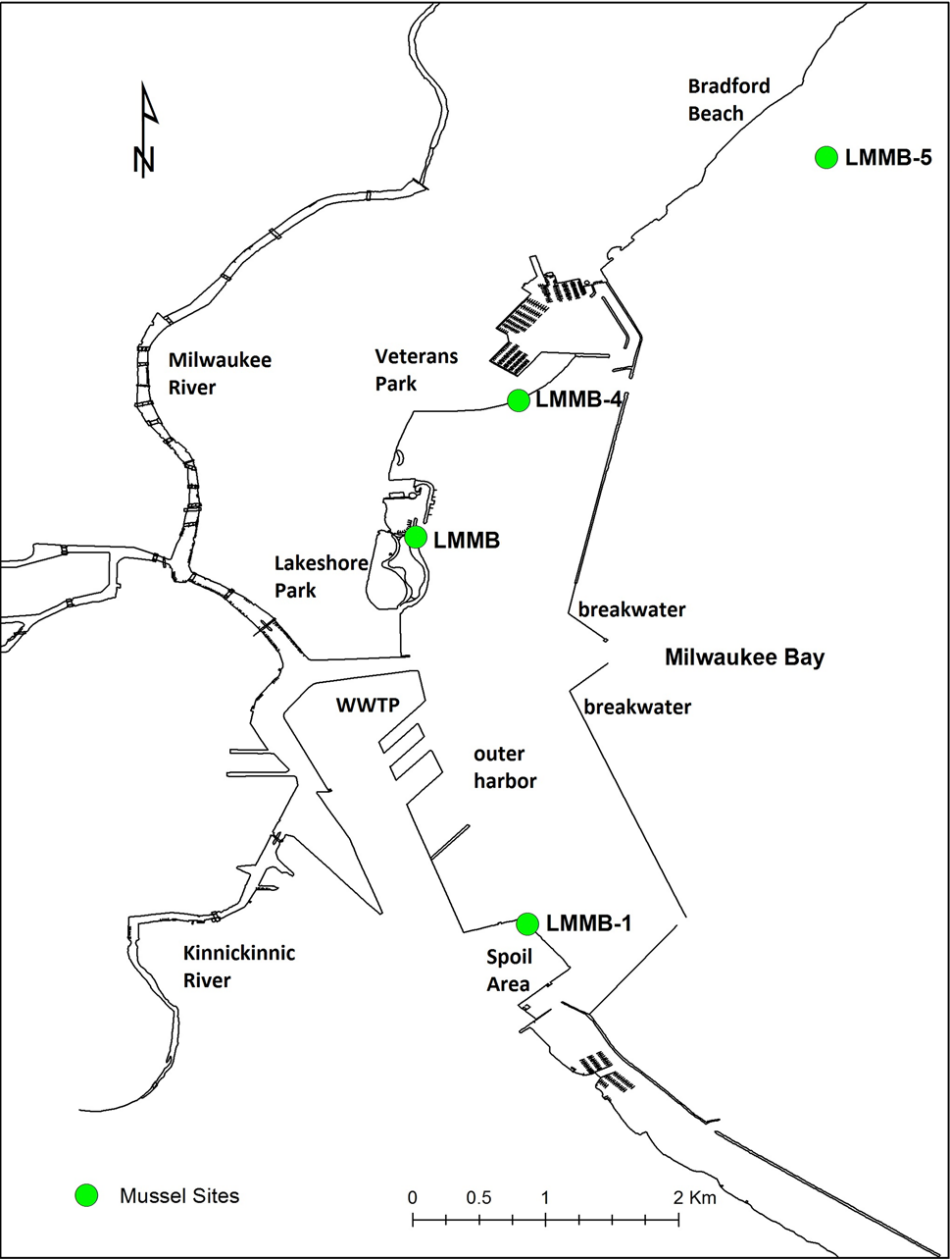
Map of Lake Michigan along the Milwaukee, WI coast indicating the sample collection sites

*Dreissena polymorpha* were collected from four stations—three harbor sites (LMMB4, LMMB1, and LMMB) in Milwaukee Estuary, and one reference site (LMMB5) in Lake Michigan, Wisconsin (Fig. 1). This coastline has been monitored for the environmental effects of historical industrial pollution as part of the Mussel Watch program (Cooksey et al. 2013; Kimbrough et al. 2008). The collection stations are near historical Mussel Watch stations, but dive sites were adjusted based on the number of mussels available on submerged stone breakwaters. Two impacted sites, LMMB4 and LMMB, are located in the outer harbor north of the harbor entrance ship channel. LMMB is adjacent to Lakeshore State Park, a 7-ha manmade island constructed mainly of dolomite limestone from a deep tunnel project of the Milwaukee Metropolitan Sewerage District in 1991. LMMB4 is adjacent to Juneau Park, whose land use has remained largely unchanged since the early 1900s. The LMMB1 site is located adjacent to the Milwaukee Harbor Confined Disposal Facility (dredge spoil) in the outer harbor south of the harbor entrance ship channel and approximately 4 km south of LMMB4. In order to assess the impact of the spatial scale within a dive station, three spots within a few meters of each other were collected and handled as distinct groups (labeled R, G or B). A reference site for this study was located in Lake Michigan approximately 0.4 km offshore of a public swimming beach and about 4 km north of the entrance to Milwaukee harbor.

The samples were collected over a 2-day period. At each station, all the mussels were collected in one dive. On a given dive station, three regions of about 25 cm diameter were sampled and these three regions were between 1 m and 4 m apart. The mussels from each of the regions were placed into a separate nylon mesh dive bag; the bags were gently shaken underwater to remove silt and debris. Mussels were removed from breakwater boulders with a sharpened stainless steel paint scraper. Upon surfacing, the harvested mussels in individual mesh bags were immediately placed in a 28 L cooler with site water (cooled by water ice in sealed plastic bags to maintain near-ambient temperatures). After transport to shore, individual mussels were separated by cutting the byssal threads with stainless steel surgical scissors. Residual silt and debris was removed from the shells with gentle rubbing under a stream of site water. The individual mussels were placed in individual Teflon bags, zip-closed with plastic cable ties holding a labeled tag and frozen by dropping into a dry shipper which had been pre-cooled with liquid nitrogen. The time between the samples breaking the surface of the water and freezing of the mussels was recorded at each collection site (LMMB: 36 min; LMMB4: 46 min; LMMB1: 49 min; LMMB5: 43 min). After shipping the frozen mussels to the analytical laboratory, the mussels were stored at −80 °C until sample processing.

Groups of approximately 20 mussels were collected from the reference site, LMMB5 and frozen together in a large Teflon bag; 15 of these bags were collected. One mussel from each bag was used in this study. Additionally, in the field, 15 individual mussels from LMMB4 were shucked, placed in individual 2 mL cryovials and snap frozen in liquid nitrogen before being placed in the dry shipper for transportation to the analytical laboratory; this set of tissues was used to produce an in-house prepared control material.

### Quality control materials

Three different materials were used for quality control during this study to demonstrate the reproducibility of the sample processing and analytical methods: In-house prepared mussel control material (MCM) and two NIST standard reference materials [SRM 1974c—Organics in Mussel Tissue (*Mytilus edulis*) (frozen mussel tissue homogenate), and SRM 2974a—Organics in Freeze-Dried Mussel Tissue (*M. edulis*)]. MCM was prepared by pooling frozen homogenized wet tissues from 10 field-shucked LMMB4 samples. The material was kept frozen while it was weighed and aliquoted by mass into bead-beating tubes (2.8 mm ceramic beads, MO BIO Laboratories). The aliquots were stored at −80 °C until use.

### Method development

A whole-body analysis approach was used in this study for a number of reasons. The small size of the zebra mussels precluded easy dissection and analysis of specific organs in the field due to the low mass of the mussels (typically less than 100 mg total tissue weight). In addition, the effects on the metabolome of dissecting the mussels in the field had not been systematically investigated so there was some advantage with a rapid-freeze protocol to arrest metabolism quickly. The snap freezing of the intact mussels proved to be important because we found that the MCM mussels which were dissected from the shell in the field present a different metabolomic profile compared to the in-shell, snap-frozen tissues (Supplemental Fig. 3).

Numerous experiments were performed to optimize the extraction of whole-body freshwater mussel materials for this study. During the method development phase, experiments were conducted to examine the differences between extractions that start with wet (frozen) tissues and dry (lyophilized) tissues. In addition, NMR sample stability at room temperature was examined based on previously reported stability issues with other whole body extractions (Liebeke and Bundy 2012).

For the wet sample versus dry sample extraction studies, a total of ten MCM samples [wet (n = 5) and dry (n = 5)], six SRM 1974c samples [wet (n = 3) and dry (n = 3)] and six SRM 2974a samples [wet (n = 3) and dry (n = 3)] were extracted. To prepare the dry tissues, MCM aliquots and SRM materials were lyophilized for approximately 15 h prior to extraction. The average water content of MCM and SRM 1974c were determined to be 88.4 and 91.1 % respectively through a gravimetric assessment of the lyophilized tissues. Though SRM 2947a is a freeze-dried sample, an average water loss of 6.1 % occurred upon lyophilization. These values were used to calculate the solvent volumes for the wet-tissue extractions in this study to compensate for the water naturally present in the tissues.

The samples were extracted (see procedure below) and subjected to NMR analysis. The samples were repeatedly analyzed over several days with the samples remaining at room temperature in the NMR auto-sampler to represent harsh sample handling conditions.

### Quality control samples

Quality control samples were processed with each batch of experimental samples and these included solvent blanks, replicate experimental sample extractions and the QC materials mentioned earlier. In total, six MCM samples and ten samples of each SRM were extracted along with the experimental samples in batch fashion. In each batch, SRM 1974c and MCM (wet homogenized tissue; 100 ± 3 mg) and SRM 2974a (lyophilized tissue; 25 ± 1 mg) were weighed into bead-beater tubes and lyophilized overnight along with the experimental samples.

### Mussel sample processing

A total of 74 individual experimental whole-body mussels were processed in this study [LMMB5 (n = 25), LMMB4 (n = 10), LMMB (n = 10), LMMB1 (n = 29)]. Mussel tissues were removed from the shells individually prior to homogenization. In order to keep mussels frozen, they were kept inside a cryogenic workstation (MVE Cryo Cart, Princeton Cryogenics, Inc.) during extraction from the shells. Using pre-cleaned surgical scalpels, the edge of the shell was cut away. Once the shell was opened, the mussel tissue was placed on a weigh boat where the mussel tissue was examined so that small pieces of shell could be gently removed using the scalpel. Any pieces of ice found in the shell were kept with the tissue to avoid any loss of tissue. Each individual mussel was homogenized using a cryogenic ball-mill homogenizer (Cryomill, Retsch Inc.) with either a 25 or 35 mL grinding jar and 15 mm and 20 mm balls, respectively, for 2 min at 25 Hz. The homogenized tissue was placed in pre-weighted 2 mL cryovials using the cryogenic workstation at each step of the sample preparation process to keep the wet tissue frozen. The experimental mussel samples were lyophilized in 2 mL cryovials for approximately 15 h, and 10 ± 1 mg of dry tissue was aliquoted into bead-beating tubes. The average tissue weights of all the test samples were used to calculate the solvent volumes for extraction. In each extraction batch, seven or eight experimental samples along with the quality control materials were extracted using a chloroform:methanol:water technique modified from Wu et al. (2008). The dried tissue samples were taken out of the −80 °C freezer right before the addition of cold solvents and the samples were kept on ice during the extraction. The ice-cold polar solvent mix [methanol (4 mL/g dry weight) (Honeywell) and Millipore water (1.6 mL/g dry weight)] was added to the tissue homogenate in the bead-beating tube. The tissue with polar solvents was mixed using a Precellys 24 homogenizer (Bertin Technologies, France) in two cycles of 15 s at 6500 rpm (681 rad/s). Using a pipette, the homogenate was transferred to a glass vial containing cold chloroform (4 mL/g wet weight) (Fisher Scientific) and Millipore water (2 mL/g wet weight) for a final solvent volume ratio of 2 chloroform:2 methanol:1.8 water. The mixture was vortexed for 30 s, and incubated on ice for 10 min. The solvent phases were partitioned by centrifugation at 2000 × *g*_n_ at 4 °C for 5 min. The upper polar phase was collected into 1.5 mL Eppendorf microcentrifuge tubes, and dried by vacuum centrifuge for 2 h at room temperature (Eppendorf, Vacufuge). The dried polar extracts were re-hydrated with 600 µL of NMR buffer containing 100 mol/L phosphate buffer, pH 7.3, 1 mol/L TMSP (3-Trimethylsilyl 2,2,3,3-d4 propionate, CAS 24493-21-8), and 1 mg/mL NaN_3_ (sodium azide CAS 26627-22-8) prepared in D_2_O, and 550 µL of each sample was then transferred into 5 mm NMR tubes (Norell).

### NMR spectroscopy

One-dimensional ^1^H NMR spectra and two-dimensional ^13^C edited heteronuclear single quantum correlation (HSQC) spectra for representative samples were acquired on a Bruker Avance II 700 MHz spectrometer equipped with a 5 mm, z-gradient TCI cryoprobe using previously published methods (Schock et al. 2013). Experiments were run with eight dummy scans (DS) and 320 acquisition scans (NS) with an acquisition time (AQ) of 2.34 s and a relaxation delay (D1) of 3.0 s for a total repetition cycle (AQ + D1) of 5.34 s. The mixing time was 60 ms. The spectral width was 20 ppm, and 64 K real data points were collected.

For HSQC data, a relaxation delay equal to 1.5 s was used between acquisitions and a refocusing delay of 3.45 ms was implemented. In general, 2048 data points in the direct dimension and 512 data points in the indirect dimension with 128 scans per increment were acquired with spectral widths of 11 ppm in F2 and 180 ppm in F1 (^13^C).

### Metabolite identification

**Fig. 2:**
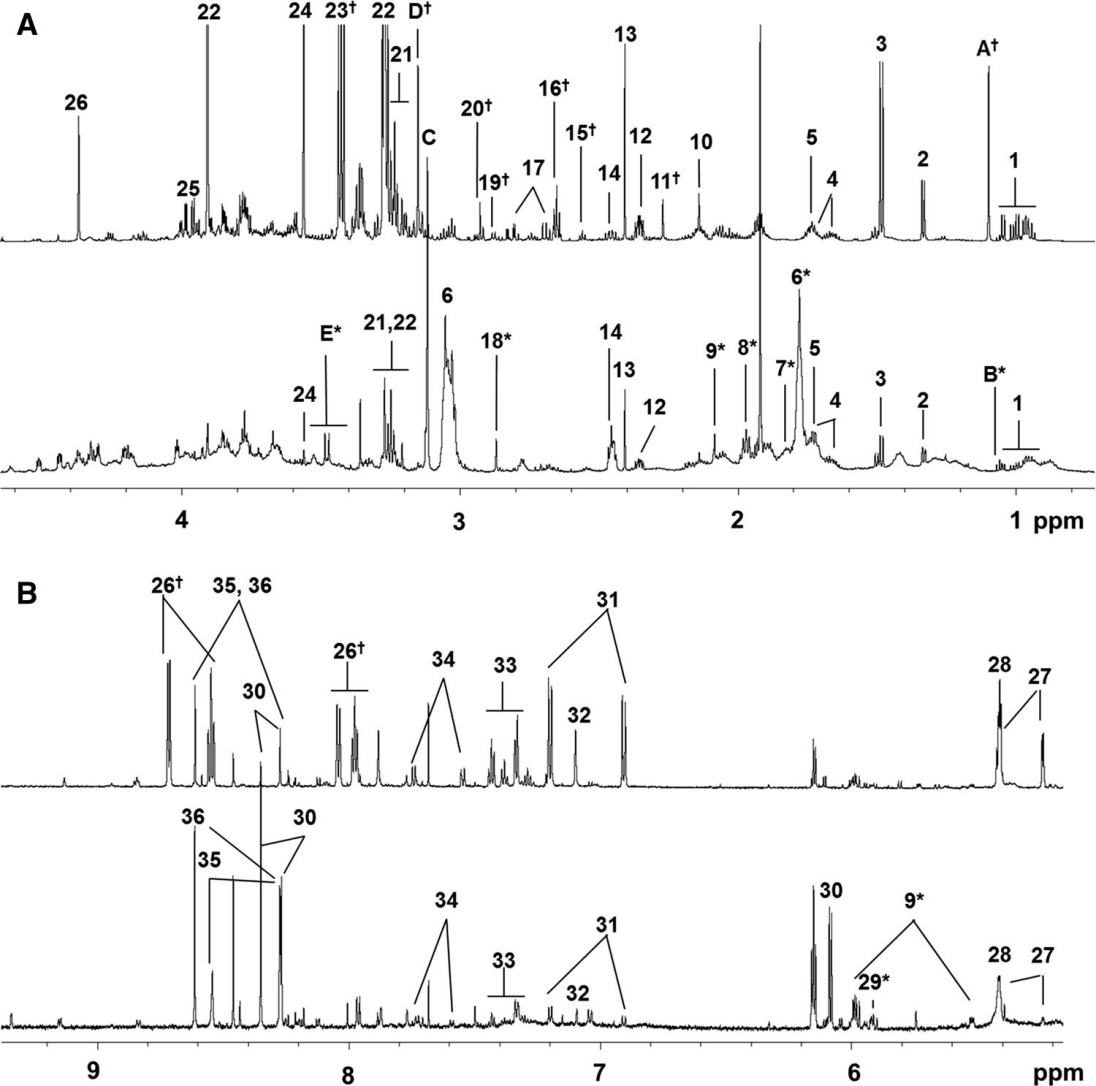
^1^H NMR spectral comparison of zebra mussel and SRM 1974c (*M. edulis*). Representative ^1^H NMR spectra of zebra mussel from the reference site (*bottom*), and *M. edulis* SRM 1974c (*top*) are shown. **a** Upfield NMR spectrum (0.0–4.0 ppm), **b** downfield NMR spectrum (5.0–10.0 ppm). (1) Leu, Ile, Val; (2) lactate, Thr; (3) Ala; (4) Arg; (5) Lys; (6) putrescine*; (7) ornithine*; (8) methyl-4-aminobutyrate*; (9) UDP-*N*-acetylglucosamine*; (10) Met; (11) acetoacetate^†^; (12) Glu; (13) succinate; (14) Gln; (15) β-Ala^†^; (16) hypotaurine^†^; (17) Asp; (18) trimethylamine*; (19) Asn; (20) l-methionine methylsulfonium iodide^†^; (21) choline, phosphocholine, glycerophosphocholine; (22) betaine; (23) taurine^†^; (24) Gly; (25) Ser; (26) homarine^†^; (27) maltose; (28) glycogen; (29) uridine*; (30) adenosine; (31) Tyr; (32) His; (33) Phe; (34) Trp; (35) ATP/ADP; (36) AMP. The *asterisks* indicates metabolites identified only in zebra mussel and (*dagger*) only in *M. edulis*. The unidentified peaks which have correlated ^13^C chemical shifts are labeled with *A*–*E*. (*A*) 1.10 ppm^†^ (s); (*B*) 1.25 ppm* (s); (*C*) 3.12 ppm (s); (*D*) 3.15 ppm^†^ (s); (*E*) 3.48 ppm* (d or two s). The complete list of annotated metabolites and their ^1^H and ^13^C chemical shifts are listed in Supplemental Tables 2 and 3

Metabolites found in zebra mussel and SRM 1974c were assigned based on 1D ^1^H and 2D ^1^H-^13^C NMR experiments. Peaks were assigned by comparing the chemical shifts and spin–spin couplings with reference spectra found in databases such as the Human Metabolome Database (HMDB) (Wishart et al. 2007), the Madison metabolomics consortium database (MMCD) (Cui et al. 2008), the biological magnetic resonance data bank (BMRB) (Ulrich et al. 2008), an in-house compiled database, the SBASE-1-1-2 and bbiorefcode_0_1_2 databases used with AMIX (version 3.9.11; Bruker Biospin, Inc., Billerica, MA), Chenomx NMR Suite profiling software (Chenomx Inc. version 7.12) and previous publications (Liu et al. 2011a, b; Wu et al. 2011; Wu and Wang 2010, 2011; Zhang et al. 2011a, b; Viant et al. 2003; Tikunov et al. 2010; Jones et al. 2008; Spann et al. 2011). Detailed assignments used for metabolite identification [Level 2, putative identification (Sumner et al. 2007)] are given in Fig. 2 and Supplemental Tables 2 and 3.

### Statistical analysis

#### Multivariate analysis

Principal components analysis (PCA) was performed to determine the reproducibility of the extraction and sample stability and to look for metabolic differences in the tissues from different collection sites. Processed ^1^H NMR spectra were binned and analyzed with AMIX for PCA analysis. The spectra from 0.5 to 10.0 ppm, excluding the region of the residual water resonance (4.7–5.0 ppm) and contaminants identified in the blanks [acetate (1.91–1.93 ppm), chloroform (7.67–7.69 ppm), and formate (8.45–8.47 ppm) (Supplemental Fig. 1)], were reduced by uniform binning to 1890 buckets 0.005 ppm wide. Signal intensities were summed for integration, and the spectra were normalized to constant total spectral area. Prior to PCA analysis, the binned spectra were mean-centered with no scaling.

In addition to the PCA analysis, additional approaches were used to analyze the multivariate results. Supervised PCA (sPCA) was used to construct hybrid loadings which optimize the separation between selected groups and aid in identification of significant classification compounds. (Rousseau et al. 2008; Schock et al. 2010) These calculations were done using Microsoft Excel.

Upon the initial PCA analysis of LMMB1 and the reference site, the variations in two buckets, 2.41 ppm [succinate Fig. 2—(13)], and 3.12 ppm [unidentified singlet (Fig. 2—(C)], were found to overwhelm the first few PCs. These two buckets were eliminated in the PCA analyses in order to detect additional components which may vary between the two groups.

#### Univariate analysis

Some data was analyzed using a univariate approach, based on bin-by-bin differences between groups of spectra from different sites. In order to identify the NMR peaks that are significantly different between the impacted sites and reference site, ^1^H significant difference spectra (SDS) were generated (Schock et al. 2012, 2013). This approach does not consider the correlation structure of the data, but provides easily interpreted difference spectra when the differences between groups are measurable. To avoid significant false discovery rates, the Student’s *t* test between groups was subjected to False Discovery Rate (FDR) control at the 0.05 level (Benjamini and Hochberg 1995). The algorithm from Sect. 3.1 of Benjamini and Hochberg (1995) was implemented in Microsoft Excel.

## Results

### Zebra mussel extraction protocol and stability

The spectra from extractions of wet and dry samples using the quality control materials (MCM, SRM 1974c and SRM 2974a) were analyzed with principal components analysis (PCA) (Supplemental Fig. 2). For the two NIST SRM samples, no significant differences between wet and dry homogenized tissue extracts were identified; these extracts were stable at room temperature with up to 4 days of room temperature storage (Supplemental Table 1). The differences between the two SRM materials, SRM 1974c and SRM 2974a, were suspected to be due to the differences in the material storage condition (wet-frozen versus freeze-dried). For MCM, on the other hand, statistically significant differences between the wet tissue and dry tissue extracts were found [PC1 (91.53 % EV), *p* = 1.48 × 10^−3^; PC2 (8.33 % EV), *p* = 1.38 × 10^−3^; PC3 (0.08 % EV), *p* = 9.89 × 10^−3^; *p* values calculated for PC scores between wet and dry MCM tissues]. The changes in the wet MCM tissue extract over time observed in the PCA scores plot (RSD = 7.39 %) were quite dramatic compared to dry MCM tissue extract (RSD = 3.39 %) and so we used the dry tissue extraction for experimental samples.

### Quality control analysis

A PCA analysis of all experimental and quality control samples shows that the repeatability of the extraction and analysis of QC materials has much less spread in the scores plot than the variability in the experimental samples (Supplemental Fig. 3A). The apparent separation between the MCM scores and the experimental mussel sample scores could reflect the field processing of the individual mussels that comprise the MCM. To assess the repeatability of the analysis for experimental samples, four experimental samples were extracted in triplicate and two experimental samples were extracted in duplicate as technical replicates (Supplemental Fig. 3B). Eight solvent blanks that did not contain any tissue were processed through the entire sample extraction process to test for unintentional contamination during processing (Supplemental Fig. 1). These spectra revealed varying levels of formate, chloroform and acetate occurring in the sample analysis, so these regions were excluded from the spectra of all analyzed samples.

In order to assess spectral repeatability, including sample processing and instrumental analysis, spectral median relative standard deviation (%RSD) (Parsons et al. 2009) was determined for each of the quality control sample spectra: SRM 1974a, SRM 2974a, MCM, and the technical replicates (Supplemental Table 1). All of the QC samples resulted in  %RSDs of less than 5 % except in the LMMB technical replicate (13.49 %). The visual inspection of NMR spectra of the LMMB samples indicated that one of the technical replicate spectra had reduced over-all signal intensity due to partial sample loss during the extraction; examination of the spectra from other samples in the affected batch indicate this was a single-sample issue. Removing this spectrum from the median  %RSD calculation resulted in a  % RSD of 4.34 %. The calculations of this QC index are very useful in identifying systematic errors during sample processing.

### Metabolic profile of zebra mussel

Overall, the concentrations of extracted metabolites from 10 mg dry tissue were significantly lower in zebra mussel samples compared to SRM 1974c. Comparing the extractable polar compounds (per g dry weight) for the zebra mussels compared to the SRM reveals that the SRM had almost 10 times the extractable polar mass compared to the zebra mussels in this study (Supplemental Table 1). This difference likely reflects a biological difference between freshwater mussels and marine mussels which could be related to ecosystem factors such as the different salinities these species experience. A total of 35 metabolites in zebra mussel and 44 metabolites in SRM 1974c were positively identified in 1D and 2D NMR spectra (Supplemental Tables 2, 3). Significant differences in metabolic profiles between marine mussels and freshwater mussels were observed in the NMR spectra (Fig. 2). Metabolites unique to zebra mussels compared to marine mussels were putresine, ornithine, trimethylamine, and methyl-4-aminobutylate. Some of the metabolites found in several species of marine mussels including SRM 1974c: homarine, hypotaruine, acetoacetate, and taurine (Viant et al. 2003; Tuffnail et al. 2009; Tikunov et al. 2010; Liu et al. 2011a), were not detected in zebra mussel. A few of the peaks previously reported as “unknowns” were also observed in zebra mussel and/or SRM 1974c (Fig. 1) (Tikunov et al. 2010; Wu et al. 2011) and remain unidentified.

### Sampling site variability

Samples collected from LMMB1 sites were compared based on the collection regions over a 1 m scale to see how sensitive the findings are based on the fine-scale sampling region choice. If significant metabolomic differences were observed on the 1 m scale, then significantly different field sampling schemes would have been designed to overcome field sample heterogeneity. The analysis included enough samples to address this field-sampling protocol issue with adequate sensitivity [LMMB1-G (n = 10), LMMB1-B (n = 9), and LMMB1-R (n = 10)]. The PCA analysis indicated that there was no apparent biological variability between the samples collected from these three regions at the LMMB1 site (Supplemental Fig. 4). The *p* values of PC1 and PC2 between the three regions were all higher than 0.05 (Supplemental Table 4A).

### Impacted sites (LMMB1, LMMB, LMMB4) versus the reference site (LMMB5)

**Fig. 3:**
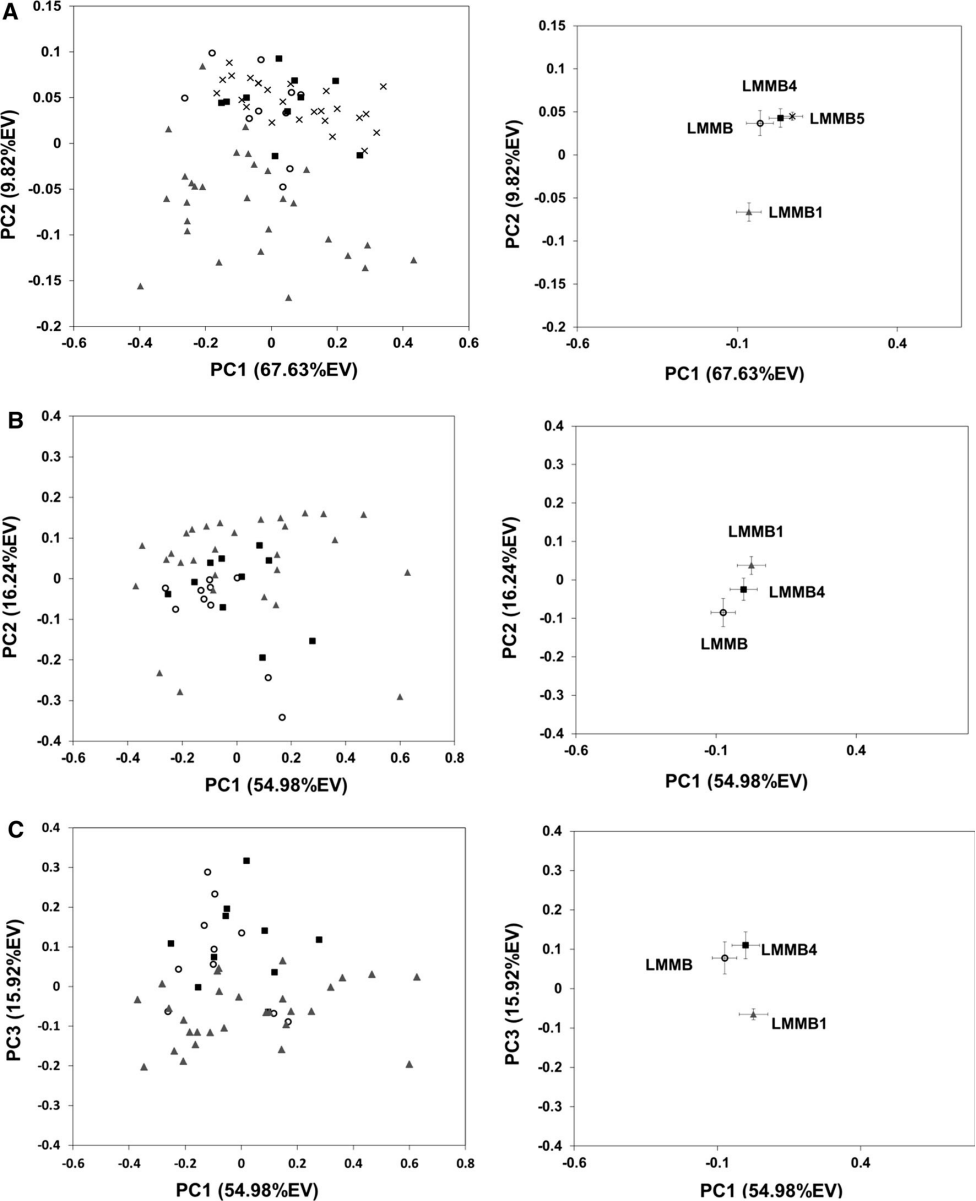
Differences between the sites. *Left* **a** PC1 vs. PC2 of PCA scores plot of all four collection sites, LMMB (*open circle*), LMMB4 (*filled square*), and LMMB1 (*filled triangle*), LMMB5 (*times*). **b** PC1 vs. PC2, **c** PC1 vs. PC3 of PCA scores plot of the zebra mussels from three impacted sites, LMMB (*open circle*), LMMB4 (*filled square*), and LMMB1 (*filled triangle*). *Right* Each point represents the mean PC score for each site. The *error bars* show ± one standard error of the mean

The first question of importance is whether there is a metabolomic difference between mussels collected at the reference site (LMMB5) and the outer harbor sites; if differences are observed, they may be due to different levels of pollution effects or to other physical differences between the sites such as water temperature, nutrient availability or turbidity levels. The PCA scores plot comparing the samples from all 4 collection sites (but no quality control samples) indicated small differences between the reference site and two sites north of the harbor ship channel, LMMB and LMMB4 (Fig. 3A; Supplemental Table 4B). However, the metabolic profile of the mussels from the south harbor impacted site (LMMB1) and the reference site show significant differences (PC1, p = 9.13 × 10^−3^; PC2, p = 1.16 × 10^−11^) when compared by PCA.

### Differences between impacted sites: (LMMB, LMMB1, LMMB4)

Another question is whether there are any differences between metabolic profiles of mussels collected at impacted sites. Samples from three impacted sites, one south (LMMB1), and two north (LMMB and LMMB4) (Fig. 1) of the harbor ship channel, were analyzed by PCA. No significant separation was observed in the PC1 and PC2 scores plot (Fig. 3B; Supplemental Table 4C). However, a significant separation was found in PC3 between the south harbor site and two north harbor sites (Fig. 3C). The *p* value of PC3 scores between LMMB1 and LMMB4 was 4.65 × 10^−4^ and LMMB1 and LMMB was 6.50 × 10^−3^. These results suggested the presence of metabolic differences in mussels from different locations within the Milwaukee Estuary. To investigate the metabolic signature that causes this difference, an sPCA hybrid loadings plot was constructed to compare between the southern south harbor site LMMB1 and two north harbor sites, LMMB and LMMB4 (Supplemental Fig. 5). This metabolic signature turned out to be very similar to the metabolic signature identified between LMMB1 and the reference site discussed below.

### Differences between the south harbor site (LMMB1) and the reference site (LMMB5)

**Fig. 4:**
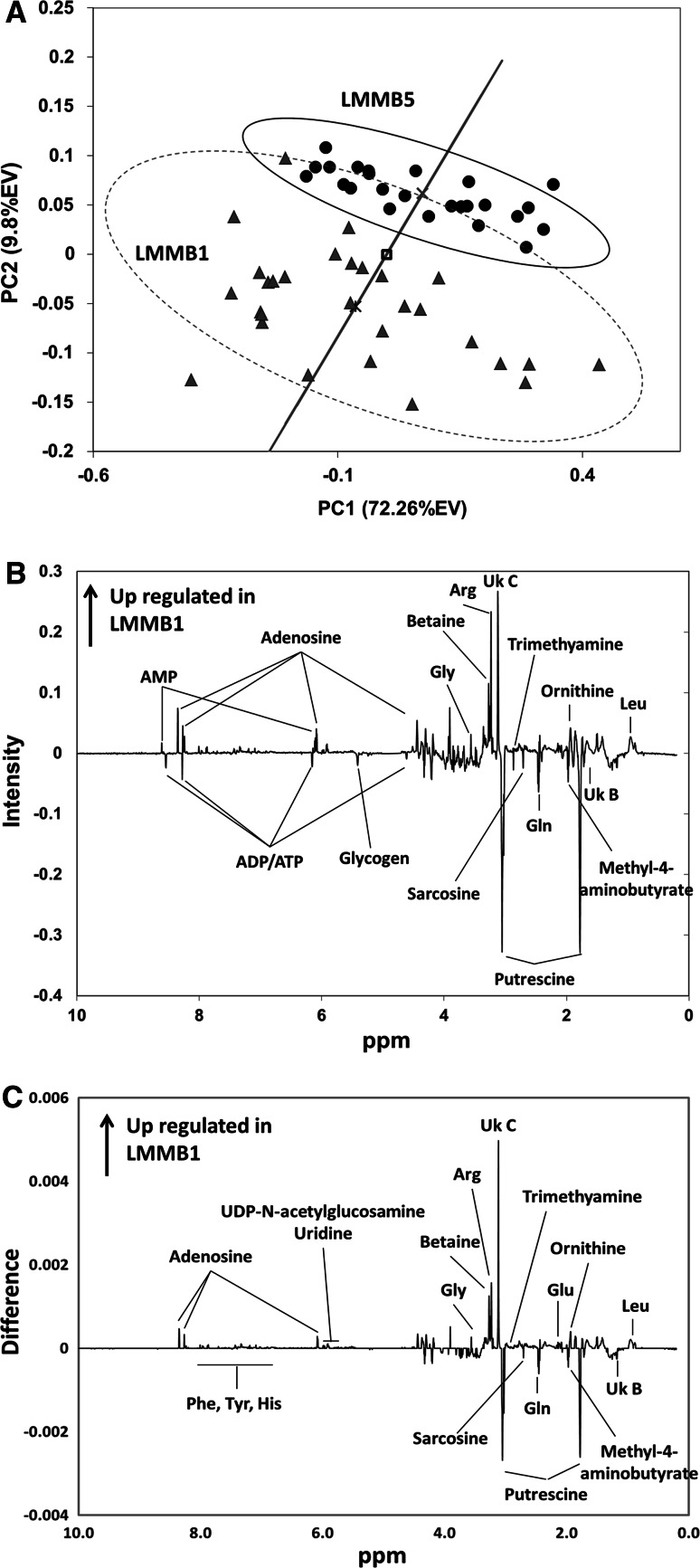
Differences between LMMB1 and the reference site. **a** PCA scores plot of the zebra mussels from impacted site LMMB1 (*filled triangle*), and the reference site LMMB5 (*filled circle*). The ovals indicate the 95 % Hoteling’s confidence interval. The *solid line* represents the projection of the scores onto the hybrid scores axis connecting the centers of each group. A Student’s *t* test for these projected points shows a significant difference between the groups (*p* = 5.65 × 10^−6^). The center of each group is represented by *times* for LMMB5 and *asterisks* for LMMB1 and *open square* for both groups. **b** A hybrid PCA loadings plot from multivariate analysis displaying the altered regions of NMR spectra between the two groups. **c** Significant difference spectra (SDS) based on Student’s *t* test and FDR correction. In both **b**, **c**, positive peaks indicate the up-regulated metabolites in LMMB1 compared to LMMB5 whereas the negative peaks are down-regulated metabolites

To examine the differences between the reference site and the south harbor site, these two sites were examined independently of the other sites. In a PCA analysis of these two sites, the clear grouping and separation of the two sites was observed in a scores plot (Fig. 4A). The separation of the two sites is significant (PC1, p = 8.23 × 10^−3^; PC2, p = 4.02 × 10^−12^). Subsequent sPCA analysis resulted in a hybrid loading plot (*p* = 5.65 × 10^−6^) (Fig. 4B) which compares favorably with a univariate analysis via SDS (Fig. 4C). These results suggest a high degree of confidence that there is a significant biological difference between the south harbor site and the reference site.

In order to identify the components in metabolic profiles responsible for separating the two sites, the NMR spectra based on the SDS plot and the sPCA hybrid loadings plot were analyzed in detail. The 300 largest magnitude spectral features in the sPCA plot were used for the identification of metabolites responsible for the separation of the two groups. In addition, the SDS spectrum was also used to identify altered metabolites based on the chemical shift of the spectral features (Table 1). The altered metabolites found in sPCA hybrid loadings were also found in the SDS analysis with few exceptions. In the SDS analysis, additional metabolites with small but significant changes were identified: phenylalanine, histidine, tyrosine, uridine, and UDP-*N*-acetylglucosamine. In the hybrid loadings plot, the increase of AMP and decrease in ATP/ADP were shown with high intensities though these buckets were not statistically significant in the univariate SDS analysis. A total of 26 altered metabolites were identified with high confidence in the comparison of zebra mussels from LMMB1 and the reference site. Two unidentified peaks at 1.25 (Uk B) and 3.12 (Uk C) ppm were also determined to be significantly altered at LMMB1. These two unknowns have previously been reported in manila clam (Wu et al. 2011), and American oysters (Tikunov et al. 2010), though their identity and role remains unknown.

**Table 1:** A table of altered metabolites identified in mussels from site LMMB1 in comparison to the reference site (LMMB5)

Metabolites	Changes^a^	Analysis^b^	Bucket^c^ (ppm)	*p* value^d^ (FDR Corr)	Metabolic pathways
Amino Acids					
Arg	↑	U/M	1.658	**3.16E**−**11**	Amino acid metabolism, urea cycle, Phosphagen biosynthesis
Gly	↑	U/M	3.563	**2.94E**−**04**	Amino acid metabolism, osmotic regulation, GSH metabolism
Leu	↑	U/M	0.953	**6.30E**−**06**	Amino acid metabolism
Gln	↓	U/M	2.468	**3.32E**−**07**	Amino acid metabolism, purine metabolism,
Glu	↑	U	2.343	**1.03E**−**05**	Amino acid metabolism, purine metabolism
His	↑	U	8.003	**6.76E**−**07**	Amino acid metabolism
Phe	↑	U	7.443	**7.62E**−**10**	Amino acid metabolism
Tyr	↑	U	6.903	**5.50E**−**04**	Amino acid metabolism
Sarcosine	↓	U/M	2.728	**6.03E**−**07**	Amino acid metabolism, osmotic regulation
Ornithine	↑	U/M	1.938	**1.87E**−**10**	Amino acid metabolism, urea cycle,
Nucleotide					
ATP/ADP	↓	M	8.548	4.89E−01	Purine metabolism
Adenosine	↑	U/M	8.358	**1.32E**−**03**	Purine metabolism
AMP	↑	M	9.613	1.52E−01	Purine metabolism
NADH	↑	U/M	8.248	**1.07E**−**03**	
Uridine	↑	U	7.873	**1.89E**−**07**	Pyrimidine metabolism
UDP-*N*-acetylglucosamine	↑	U	7.953	**6.48E**−**07**	
Carbohydrates					
Glycogen	↓	M	5.418	4.17E−01	
Osmolytes/organic compounds					
Betaine	↑	U/M	3.273	**8.67E**−**12**	Amino acid metabolism, osmotic regulation
Trimethylamine	↓	U/M	2.878	**1.24E**−**02**	
Putrescine	↓	U/M	3.053	**1.40E**−**03**	Polyamine biosynthesis
Choline	↑	U/M	3.208	**8.02E**−**04**	Glycerophospholipid metabolism
Glycerophosphocholine	↑	U/M	3.223	**2.68E**−**08**	Glycerophospholipid metabolism
Phosphocholine	↑	U/M	3.228	**1.67E**−**07**	Glycerophospholipid metabolism
Other					
Methyl-4-aminobutyrate	↓	U/M	1.973	**2.35E**−**04**	
Unknown B	↓	U/M	1.258	**3.03E**−**06**	
Unknown C	↑	U/M	3.118	**8.60E**−**03**	

Significant changes are shown in bold

^a^The symbol ↑ indicates increased levels at the LMMB1 site compared to the reference site (LMMB5)

^b^The type of statistical analysis used to identify the altered metabolites *U*—univariate and *M*—multivariate analysis

^c^The chemical shift of the bucket used for the p value calculation

^d^The FDR corrected alpha value for the corresponding buckets

### Biological significance

**Fig. 5:**
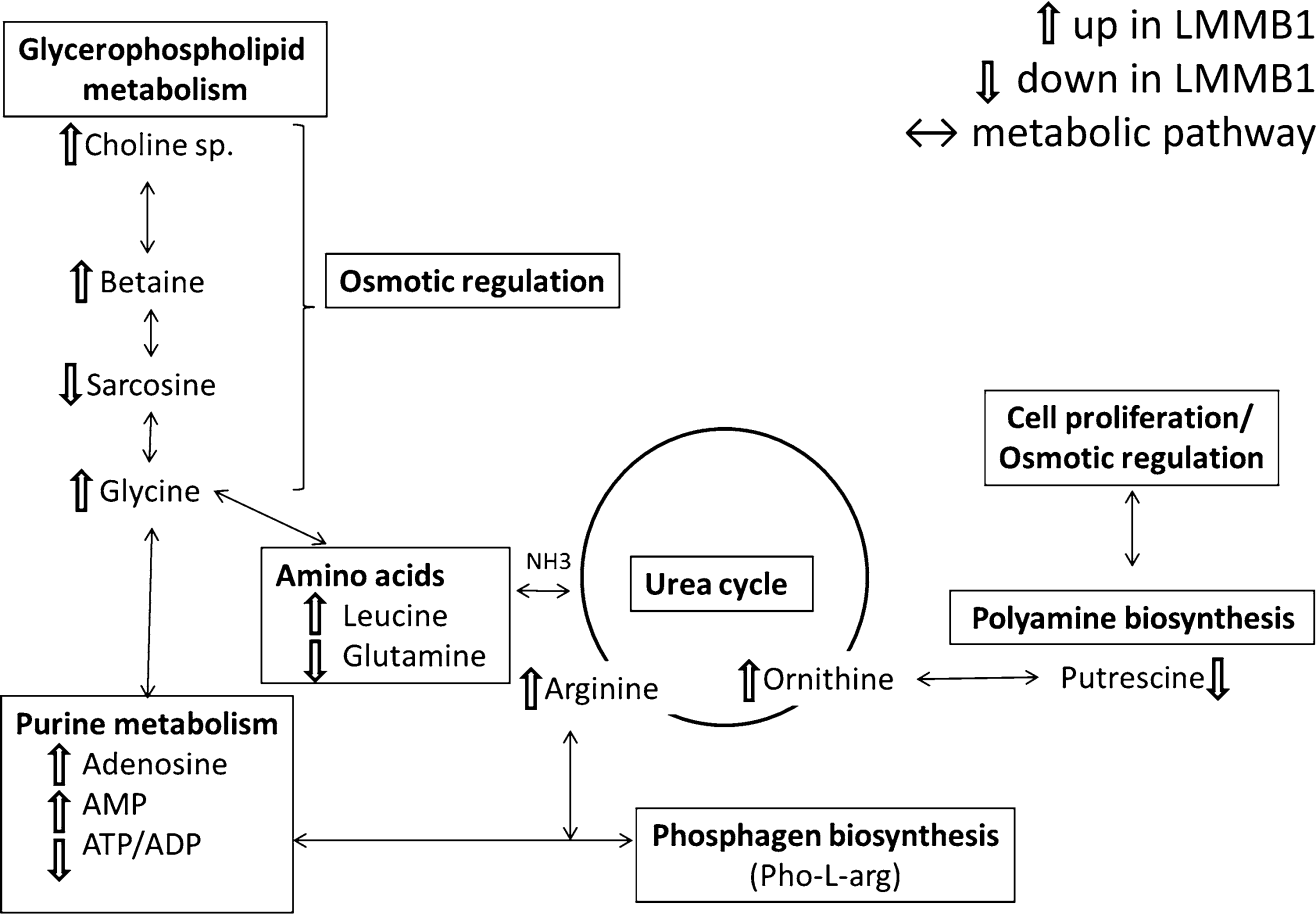
Altered biochemical pathways in zebra mussels from the southern harbor site (LMMB1) compared to the LMMB5 reference site

Though zebra mussels have been extensively used as a bio-indicator species to monitor environmental conditions, little is known of their metabolism due to toxicity studies concentrating on only a few biochemical pathways. Based on the information available from published work on marine mollusks, some of the altered metabolites identified in a comparison of mussels from LMMB1 to mussels from the reference site, LMMB5, can be considered. Our results suggest that the south harbor site (LMMB1) is more impacted compared to the reference site (Fig. 4A) and no significant signs of impact were identified from two north harbor sites (Fig. 3A). A total of 26 metabolites were identified in the comparison between LMMB1 and LMMB5 (Table 1), which were similar to the differences between LMMB1 and other two harbor sites, LMMB and LMMB4 (Supplemental Fig. 5). The metabolic pathways which are potentially altered in the mussel from the impacted site were identified using the KEGG database (Kanehisa 2002). Many of the metabolites are involved in multiple metabolic pathways; only the pathways with more than one altered metabolite are shown in Fig. 5 and discussed.

Out of 26 altered metabolites, the most apparent differences between the mussels from impacted and reference sites were in the levels of amino acids. A total of 10 altered amino acids were identified by this analysis. Changes in amino acid concentrations as a response to general stress have been documented (Jones et al. 2008; Connor et al. 2004). Several additional studies have identified alterations in amino acid concentrations in response to toxicant (mainly heavy metals) exposure in marine mollusks (Liu et al. 2011a, b; Wu et al. 2011; Viant et al. 2003; Spann et al. 2011). Altered levels of the organic osmolytes glycine and betaine may be due to a disturbance of osmotic regulation in mussels at the LMMB1 site (Fig. 5). The common marine organism osmolytes, homarine and taurine, were not detected in zebra mussel (Preston 1993; Liu et al. 2011b). Altered levels of choline (*p* = 8.02 × 10^−4^), phosphocholine (*p* = 1.67 × 10^−7^), and glycerophosphocholine (*p* = 2.68 × 10^−8^) suggested altered glycerophospholipid metabolism which could be caused by disturbed cellular membranes. These findings suggest changes in osmotic regulation of the freshwater mussels in a highly contaminated environment, consistent with a preliminary sediment toxicity report that indicated that the area around LMMB1 has higher sediment toxicity than LMMB4 and LMMB (Cooksey et al. 2013).

The altered level of several nucleotides in mussels from the LMMB1 site indicate potential nucleotide metabolism disturbance. A number of environmental stresses have been shown to influence nucleotide metabolism. In mammalian systems, altered nucleotide metabolism has been shown to correlate with peroxisome proliferation (Ringeissen et al. 2003). Perhaps peroxisome proliferation in the LMMB1 site mussels is induced to transport toxic substances out of the system. On the other hand, exposure to toxicants has been shown to induce catalysis of phosphoarginine which is the primary phosphagen in invertebrates because of the greater energy demands of the stress response. Previously, this process was demonstrated in red abalone where the phosphate from phosphoarginine was transferred to ADP to produce ATP and arginine in order to maintain a stable ATP source (Viant et al. 2001, 2003). In this study, higher levels of adenosine and AMP along with a decrease in ATP/ADP were identified in the mussels from the impacted site (Fig. 5). Alteration of ATP/ADP levels along with arginine has been reported in several studies in marine mollusks under different types of stress (Jones et al. 2008; Spann et al. 2011; Viant et al. 2003; Liu et al. 2011b). Arginine is a guanido compound of prime importance in all mollusks providing the phosphagen, Phos-l-arg (Thoai and Roche 1960; Gasparini and Audit 2000). In addition to the alteration in nucleotides, a significant increase in the arginine level (*p* = 3.15 × 10^−11^) was found in the mussels from the LMMB1 site. In these previous published studies, the directions of alteration in adenosine nucleotides, ATP/ADP, and arginine concentration in response to various stresses are not consistent. This may be due to the differences in species and/or type of stresses. Nonetheless, our results of the altered levels of adenosine nucleotides and arginine suggest altered phosphagen metabolism along with altered purine metabolism in mussels from the impacted site.

Putrescine is one of the polyamines, which are organic cations that interact with negatively charged molecules such as nucleic acids and phospholipids in the cell (Janne et al. 2004). Since these polyamines are involved in stability of DNA structure during replication and cell proliferation, the reduction of polyamine biosynthesis and induction of catabolism halts cell growth. A study has shown that catabolism is the overriding control mechanism of polyamine metabolism in mammals (Suppola et al. 2001). Others have reported decreased putrescine in response to Zn and/or Cd exposure in Asian clam (Spann et al. 2011) and the alteration of polyamine metabolism by osmotic and nutritional stress has been demonstrated in blue crab (Lovett and Watts 1995), white shrimp (Schock et al. 2013), brine shrimp (Watts et al. 1996) and bivalve mollusk (Gasparini and Audit 2000). In addition, putrescine has been shown to affect the ATP/ADP levels by inhibition of Na^+^/K^+^-ATPase during the regulation of osmotic and ionic homeostasis (Lovett and Watts 1995). From the results of this study, it was not possible to distinguish whether the cause of the significant decrease in putrescine level (*p* = 1.40 × 10^−3^) at the LMMB1 site was due to the reduction of polyamine biosynthesis or due to increased catabolism. Though the other polyamines, spermidine, and spermine, were not detectable in this analysis, the significant increase in the metabolites ornithine (*p* = 1.87 × 10^−10^) and arginine (*p* = 3.15 × 10^−11^) at the LMMB1 site (both are precursors of putrescine metabolism) were detected (Fig. 5). Taken together, these findings support that the down regulation of putrescine may be caused by induced polyamine catabolism under environmental stress at highly impacted sites, which may be another contributing factor to the altered level of energy metabolites: ATP and/or ADP.

## Summary

A significant challenge in metabolomics research on organisms collected from their natural habitat is the difficulty in pinpointing specific causes of metabolome alteration because so many factors occur which cannot be controlled as well as in a laboratory-based experiment. A myriad of factors like water quality, temperature, pH, toxicant exposure and food sources may produce changes in the metabolomic profile. The response to such environmental variables may themselves be dependent on life stage, sex, size or age of the individual animal. In this study, consistent field-collection protocols and a short sampling period reduced the effects of environmental and biological variability, and mussel samples from two distinct locations in the Milwaukee Estuary were distinguished by their metabolic profiles. Whole-body metabolic profiles of individual zebra mussels were analyzed using NMR-based metabolomics and the metabolic differences of mussels collected at various sites were identified. Out of three harbor sites, mussels collected from south harbor showed significant differences from the reference site (LMMB5), whereas no significant differences between the mussels from north harbor and the reference site were observed. This finding was in agreement with the preliminary sediment toxicity report that indicated that the area around LMMB1 has higher sediment toxicity than LMMB4 and LMMB (Cooksey et al. 2013). Clear distinctions were observed between the reference site (LMMB5) and the inner harbor sites, with one site (LMMB1) being notably different.

A total of 26 altered metabolites (including two unidentified peaks) were successfully identified in a comparison of zebra mussels from the LMMB1 site and LMMB5 reference site. The application of both uni- and multivariate analysis not only confirmed the variability of altered metabolites but also ensured that these metabolites were identified via unbiased analysis. The ability to detect these affected metabolites without eliminating other potential factors such as tissue type, sex, and size of the animal emphasize that a whole-body NMR-based metabolomics approach is a powerful tool for evaluation of toxicity in the environment. Due to the limited metabolic knowledge of freshwater mussels, additional work is needed to confirm the precise cause of these metabolic alterations. The identification of these altered metabolites will contribute to the future development of in situ environmental monitoring systems at impacted sites using abundant indicator organisms.

In conclusion, this study has demonstrated the feasibility of an NMR-based metabolomics approach in identifying the physiological impact of toxicant exposure in zebra mussels. The development of metabolomic fingerprints of an important freshwater species will contribute to the future investigation of environmental studies in freshwater systems. Moving forward, the metabolic profiles of zebra mussels from sites with various levels of impacts and a reference site will provide us an additional bio-indicator to monitor health and recovery in the Great Lakes.

## Electronic supplementary material

Below is the link to the electronic supplementary material.

Supplementary material 1 (DOCX 483 kb)
