# Supplementary material for: Application of NMR-based metabolomics for environmental assessment in the Great Lakes using zebra mussel (Dreissena polymorpha)
Source: Metabolomics. 2015 Feb 21;11(5):1302–15. doi: 10.1007/s11306-015-0789-4 (PMC4559106; doi:10.1007/s11306-015-0789-4)
Supplement: Supplementary file 1 — Supplementary material 1 (DOCX 483 kb) [file 11306_2015_789_MOESM1_ESM.docx]

**Supplemental Materials for:** Application of NMR-based Metabolomics for Environmental Assessment in the Great Lakes using Zebra Mussel (*Dreissena polymorpha*)

**Authors:** Miki Watanabe^1^, Kathryn A Meyer^1^, Tyler M. Jackson^1^, Tracey B. Schock^1^, Ed Johnson^2^, Daniel W. Bearden^1*^

1. Chemical Sciences Division, National Institute of Standards and Technology, Hollings Marine Laboratory, 331 Ft. Johnson Rd., Charleston, SC, United States of America,

2. NOAA Mussel Watch Program, National Oceanic & Atmospheric Administration, National Centers for Coastal Ocean Science, 1305 East West Highway, SSMC4, Room 9202, Silver Spring, MD 20910

* Corresponding Author: [dan.bearden@nist.gov](mailto:dan.bearden@nist.gov)

**Content:**

**Supplemental Figure 1.** **1D ^1^H NMR spectra of extraction blanks (bottom seven) and the NMR buffer (top)**.

**Supplemental Figure 2. NMR sample stability in wet and dry extracts from quality control materials.**

**Supplemental Figure 3. Quality assessments of the extraction method**.

**Supplemental Figure 4. Differences between three collection sites in LMMB1 1 m apart.**

**Supplemental Figure 5. A hybrid sPCA loadings plot.**

**Supplemental Table 1. Median %RSD values calculated for method development samples, QC samples, and replicate analysis samples.**

**Supplemental Table 2. The list of level 2 metabolite identifications (Zebra Mussel).**

**Supplemental Table 3. The list of level 2 metabolite identifications (SRM 1974c).**

**Supplemental Table 4. Significance Table.**

**Supplemental Figure 1.** **1D ^1^H NMR spectra of extraction blanks (bottom seven) and the NMR buffer (top)**. The arrows indicate the regions excluded in the Principal Component Analyses: 1) acetate (1.91 ppm -1.93 ppm), 2) water (4.7 ppm -5.0 ppm), 3) chloroform (7.67 ppm -7.69 ppm), 4) formate (8.45 ppm -8.47 ppm)

**
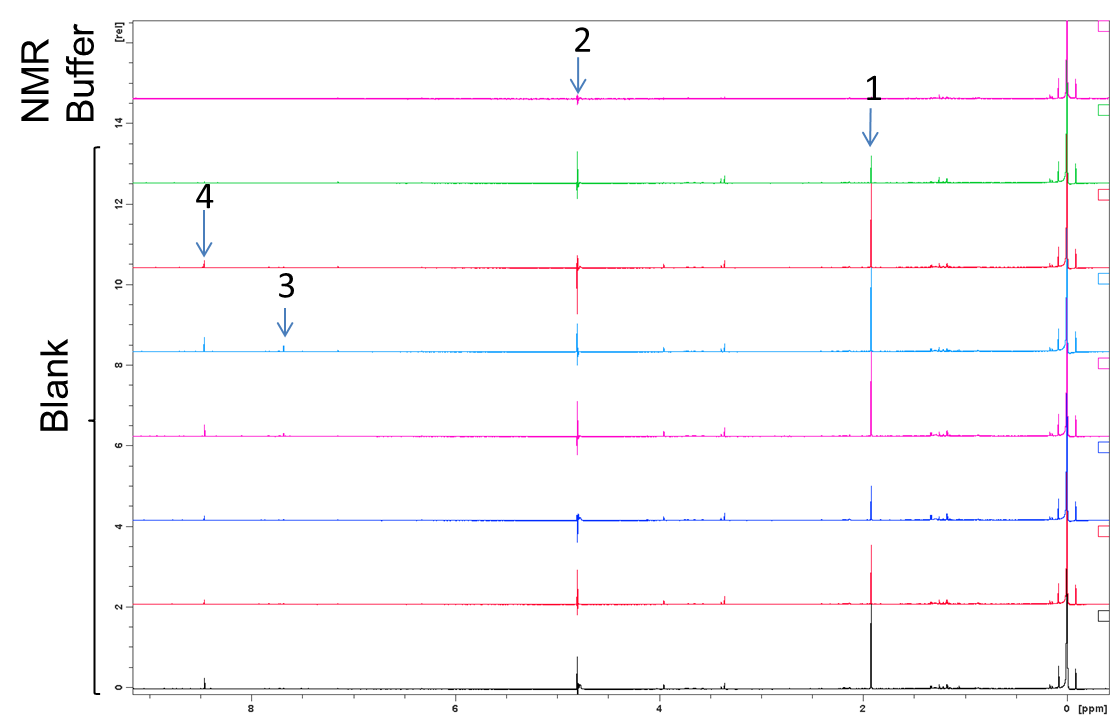
**

**Supplemental Figure 2. NMR sample stability in wet and dry extracts from quality control materials.** PCA scores plot of a representative NMR samples (6 individual samples) from wet (● blue) and dry (● red) mussel control material (MCM), wet (▲ purple) and dry (∆ orange) SRM 1974c, and wet (■ green) and dry (□ pink) SRM 2974a. The points represent the data collected at 0 hours, 15 hours , 27 hours, 3 days and 4 days from the initial data collection. The spread of the data points in wet MCM samples indicates the changes in the sample over time.


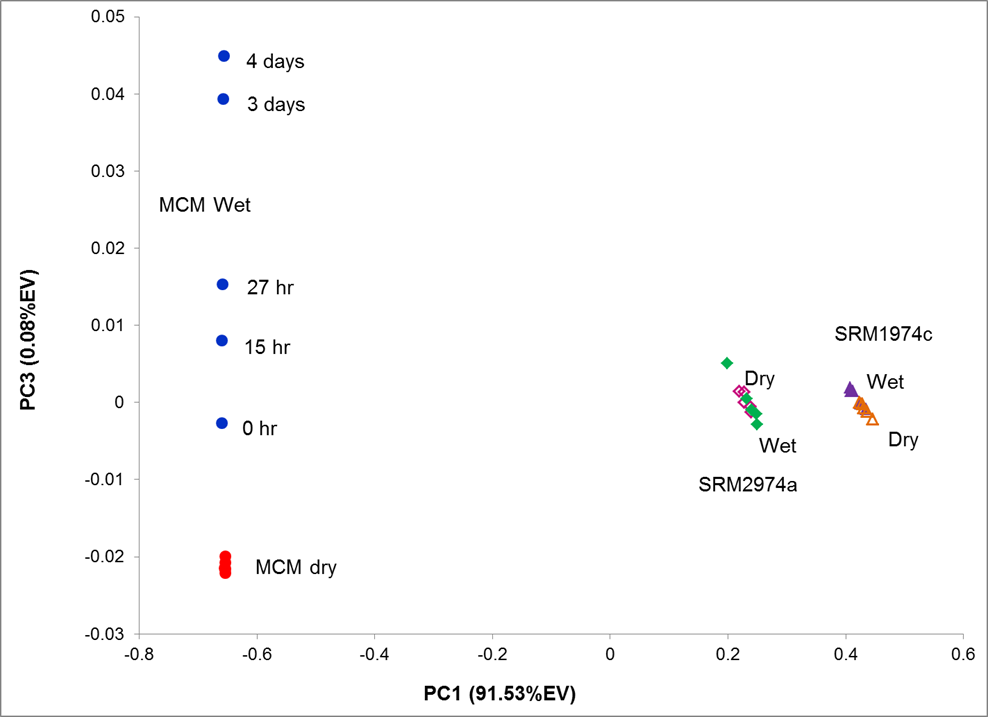


**Supplemental Figure 3. Quality assessments of the extraction method**. (A) PCA scores plot of all the mussel test samples (● black), mussel control material (MCM) (⬩ blue), SRM 1974c (■ green), and SRM 2974a (▲ red) indicating the minimal extraction variability between the extraction sets. (B) PCA scores plot of the technical replicates of the mussel test samples (○), MCM (⬩ blue), SRM 1974c (■ green), and SRM 2974a (▲ red). A total of six test samples were extracted in triplicates or duplicates and each replicate sample is shown in different colors.
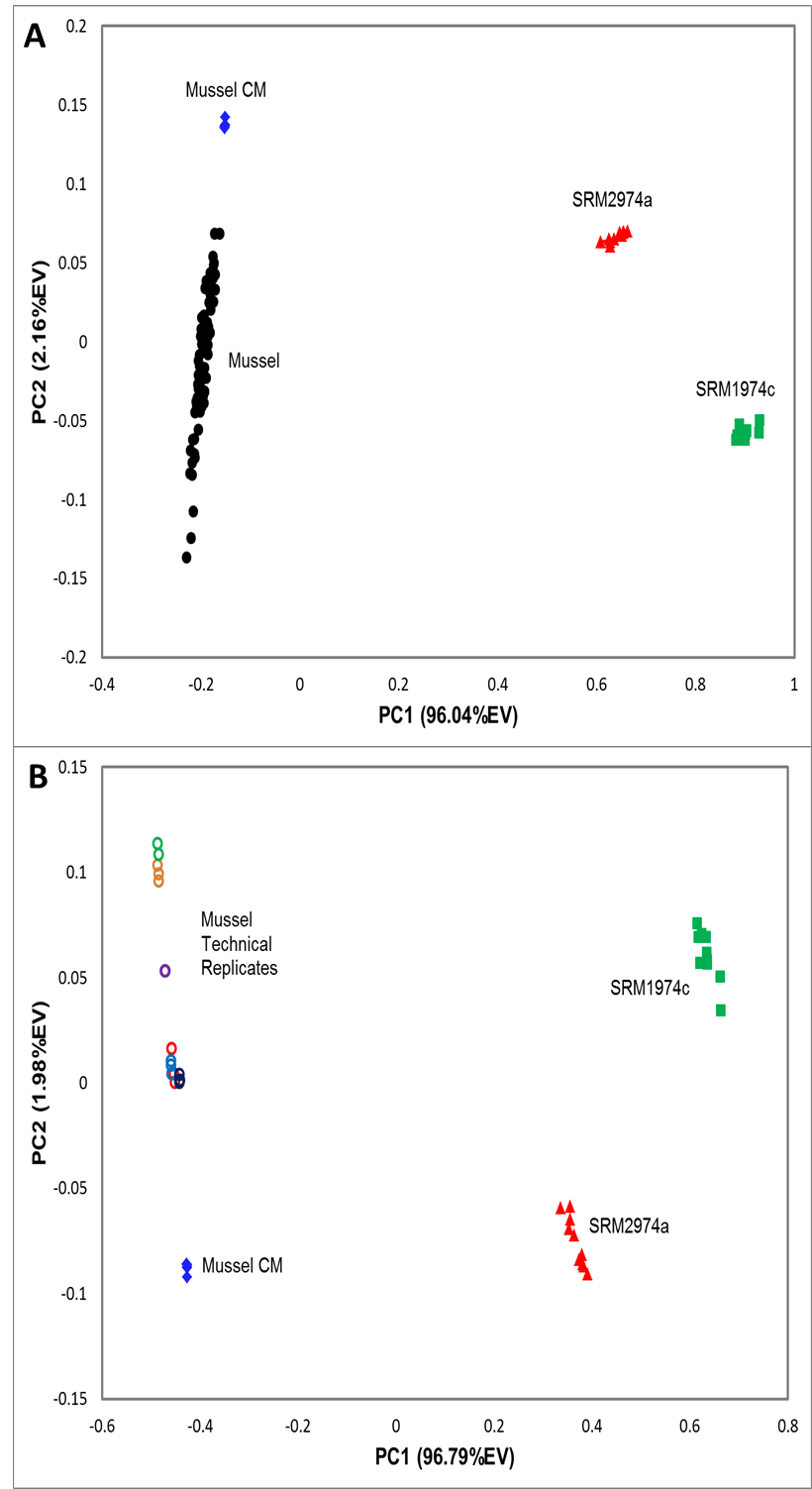


**Supplemental Figure 4. Differences between three collection sites in LMMB1 1 m apart.** (A) PC1 vs.PC2 of PCA scores plot of the zebra mussels from three collection site within LMMB1, LMMB1G (○), LMMB1B (■), and LMMB1R (▲). (B) Each point represents the mean PC score for each collection site. The error bars show +/- one standard error of the mean.

**
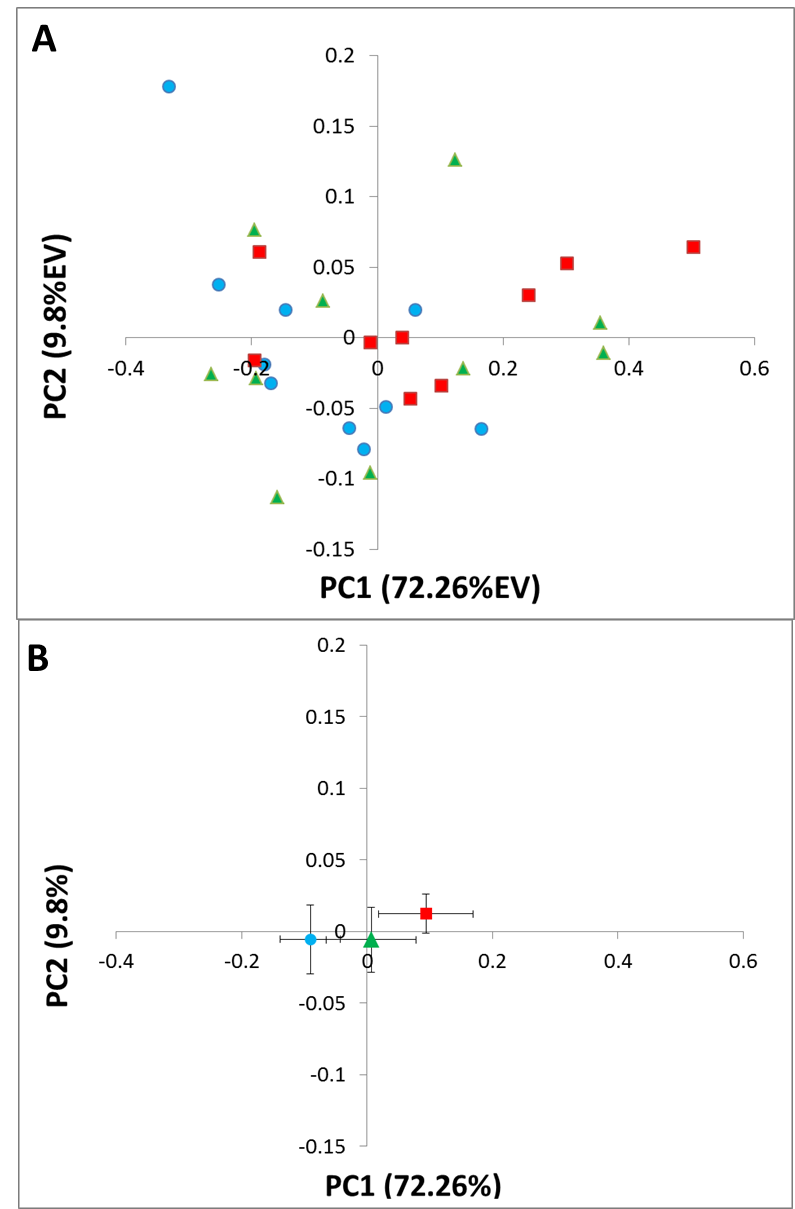
Supplemental Figure 5.** A hybrid sPCA loadings plot from multivariate analysis displaying the altered regions of NMR spectra between the southern inner harbor site LMMB1 and two sites north of river outlet, LMMB and LMMB4.

**
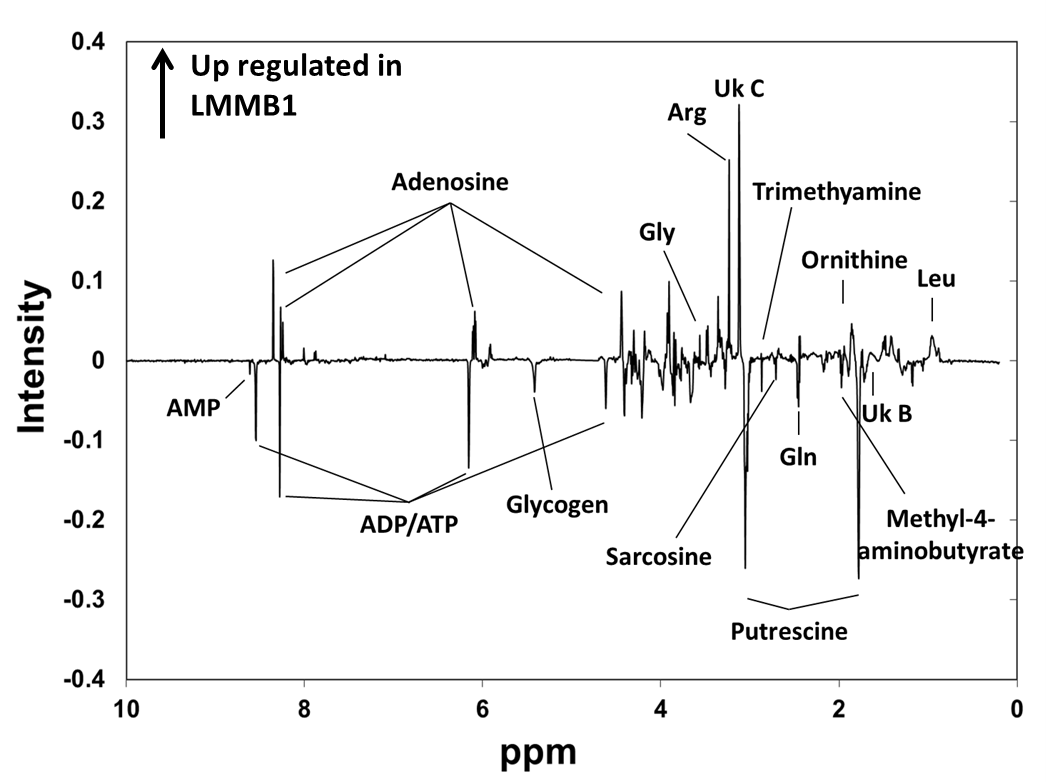
**

**Supplemental Table 1.** Median %RSD values calculated for method development samples, QC samples, and replicate analysis samples. Number of samples used during the study are listed (N) along with their % RSD. The average extractable dry metabolites were obtained during the method development. The %RSD indicating the sample stability was calculated from one of the representative samples from each type of QC sample.

| **Sample** |  | **(N)** | **%RSD** | **Average extractable dry polar metabolites (mg/g dry weight)** | **Sample stability study (number of time points)** | **Sample stability (%RSD)** |
| --- | --- | --- | --- | --- | --- | --- |
| Method | MCM wet | 5 | 5.76 | 0.0353 | 5 | 7.39 |
| development | MCM dry | 5 | 5.11 | 0.0366 | 5 | 3.39 |
|  | SRM 1974c wet | 3 | 3.03 | 0.2704 | 5 | 2.6 |
|  | SRM 1974c dry | 3 | 5.69 | 0.2055 | 5 | 2.71 |
|  | SRM 2974a wet | 3 | 4.35 | 0.1907 | 5 | 2.39 |
|  | SRM 2974a dry | 3 | 4.47 | 0.1685 | 5 | 2.88 |
| QC batch results | SRM 1974c (dry) | 10 | 4.63 |  |  |  |
|  | SRM 2974a (dry) | 10 | 3.78 |  |  |  |
|  | MCM (dry) | 6 | 4.56 |  |  |  |
| Technical Replicates | LMMB1 | 3 | 4.86 |  |  |  |
|  | LMMB | 3 | 13.49 |  |  |  |
|  | LMMB^a^ | 2 | 4.34 |  |  |  |
|  | LMMB4 | 3 | 4.67 |  |  |  |
|  | LMMB5_A | 3 | 5.63 |  |  |  |
|  | LMMB5_B | 2 | 4.28 |  |  |  |
|  | LMMB5_C | 2 | 4.29 |  |  |  |

^a^ One LMMB sample was identified as a faulty extraction and removed from the calculation.

**Supplemental Table 2.** The list of level 2 metabolite identifications *^(^*[*^31^*](#_ENREF_31)*^)^* and their ^1^H and ^13^C chemical shifts in ppm in zebra mussel.

| **Annotation** | **1H** | **13C** | **Data base (Ref) ^b^** | **Fig.2** | **Annotation** | **1H** | **13C** | **Data base (Ref) ^b^** | **Fig.2** |
| --- | --- | --- | --- | --- | --- | --- | --- | --- | --- |
| **AMP** | 8.61 |  |  | 36 | **Lactate** | 1.33 | 22.68 | HMDB01311 | 2 |
|  | 4.38 | 88.01 | HMDB00045 |  | **Leucine** | 0.97 | 25.03 | HMDB00687 | 1 |
| **ADP** | 4.77 | 77.15 | HMDB01341 |  |  | 0.96 | 23.87 |  |  |
| **ATP/ADP** | 8.54 |  |  | 35 | **Lysine** | 1.90 | 32.75 | HMDB00182 | 5 |
| **AXP** | 6.16 | 89.82 |  |  |  | 1.71 | 29.18 |  |  |
| **AXP/Adenosine** | 8.28 | 155.92 |  |  | **Maltose** | 5.24 |  |  | 27 |
| **Adenosine** | 8.35 | 143.57 | HMDB0050 | 30 |  | 4.66 | 99.05 | HMDB00163 |  |
|  | 6.09 | 91.53 |  |  | **Methyl-histidine** ^a^ | 8.01 |  |  |  |
|  | 4.44 | 73.75 |  |  |  | 7.04 |  |  |  |
|  | 4.30 | 89.14 |  |  | **N-acetylornithine** | 4.17 | 57.97 | MMCD exp00184 |  |
|  | 3.91 | 64.39 |  |  |  | 2.04 | 25.19 |  |  |
|  | 3.85 | 64.47 |  |  |  | 1.72 | 31.35 |  |  |
| **Alanine** | 1.49 | 19.06 | HMDB00161 | 3 | **NAD+** | 9.34 |  |  |  |
| **Anserine** | 3.67 | 35.11 | HMDB00194 |  |  | 9.15 |  |  |  |
|  | 3.19 | 38.37 |  |  |  | 8.84 |  |  |  |
| **Arginine** | 3.25 | 43.45 | HMDB 00517 | 4 |  | 8.19 |  |  |  |
|  | 1.90 | 30.76 |  |  |  | 6.04 | 89.45 | HMDB00902 |  |
|  | 1.66 | 26.64 |  |  |  | 4.62 | 73.24 |  |  |
| ***** | 3.77 | 57.35 |  |  | **NADH** | 8.24 |  |  |  |
| **Aspartate** ^a^ | 2.81 |  |  | 17 |  | 6.12 | 89.70 | MMCD_nmr00098 |  |
|  | 2.68 |  |  |  | **NADH/NAD+** | 8.43 |  |  |  |
| **Betaine** | 3.90 | 69.37 | HMDB00043 | 22 | **Ornithine** | 1.93 | 30.47 | HMDB00214 | 7 |
|  | 3.27 | 56.42 |  |  |  | 1.83 | 24.79 |  |  |
| **Choline** | 4.12 | 57.92 | HMDB00097 | 21 | ***** | 3.77 | 57.35 |  |  |
|  | 3.20 | 56.80 |  |  | ***** | 3.05 | 41.61 |  |  |
| **Carnosine** | 4.33 | 56.44 | HMDB00033 |  | **Phosphocholine** | 3.23 | 57.34 | HMDB01565 | 21 |
|  | 3.26 | 38.45 |  |  | **Putrescine** | 1.78 | 26.68 | HMDB01414 | 6 |
|  | 2.77 | 34.80 |  |  | ***** | 3.05 | 41.61 |  |  |
| **Glutamate** | 2.35 | 36.48 | HMDB00148 | 12 | **Sarcosine** | 2.71 | 35.32 | HMDB00271 |  |
|  | 2.06 | 29.85 |  |  | **Succinate** | 2.41 | 37.06 | HMDB00254 | 13 |
| **Glutamine** | 2.46 | 35.03 | HMDB00641 | 14 | **Threonine** | 1.34 | 23.11 | HMDB00167 | 2 |
|  | 2.14 | 28.93 |  |  | **Trimethylamine** | 2.87 | 45.39 | HMDB00906 | 18 |
| ***** | 3.77 | 57.35 |  |  | **UDP-N-acetylglucosamine** | 5.99 | 91.48 | HMDB00290 | 9 |
| **Glutathione** | 3.77 | 46.43 | HMDB00125 |  |  | 5.97 | 105.73 |  |  |
|  | 2.17 | 28.28 |  |  |  | 4.37 | 76.85 |  |  |
| **Glycogen /Maltose** | 5.41 | 102.66 | HMDB00757 | 28 |  | 4.30 | 86.35 |  |  |
| ***** | 3.96 | 76.39 |  |  |  | 4.23 | 67.96 |  |  |
| ***** | 3.82 | 63.48 |  |  |  | 3.98 | 57.73 |  |  |
| ***** | 3.79 | 63.55 |  |  |  | 3.86 | 63.46 |  |  |
| ***** | 3.72 | 75.72 |  |  |  | 2.09 | 25.04 |  |  |
| ***** | 3.66 | 79.87 |  |  | **Valine** | 1.00 | 19.50 | HMDB00883 | 1 |
| ***** | 3.65 | 74.63 |  |  |  | 1.05 | 20.79 |  |  |
| ***** | 3.43 | 72.44 |  |  | **methyl_4_aminobutyrate** | 3.69 | 56.59 | MMCD_exp00179 | 8 |
| **Glycerophosphocholine** | 3.23 | 56.99 | HMDB00086 | 21 | ***** | 3.05 | 41.61 |  |  |
| **Glycine** | 3.56 | 44.79 | HMDB00123 | 24 |  | 2.54 | 34.41 |  |  |
| **Histidine** ^a^ | 7.77 |  |  |  |  | 1.97 | 25.87 |  |  |
|  | 7.09 |  |  |  | **Unknown B** | 1.25 |  |  | B |
| **Isoleucine** | 0.94 | 14.01 | HMDB00172 | 1 | **Unknown C** | 3.12 | 55.71 |  | C |
|  |  |  |  |  | **Unknown E** | 3.48 | 54.53 |  | E |
|  |  |  |  |  |  | 3.47 | 54.36 |  |  |

* Resonance with multiple metabolite overlaps

^a^ Metabolites that could not be confirmed by the ^13^C chemical shifts were identified using Chenomx database

^b^ Database and metabolite ID used to confirm the metabolites identification

**Supplemental Table 3.** The list of level 2 metabolite identifications and their ^1^H and ^13^C chemical shifts in ppm in SRM 1974c

| **Annotation** | **^1^H** | **^13^C** | **Data base (Ref)^b^** | **Fig.2** | | **Annotation** | **^1^H** | **^13^C** | **Data base (Ref) ^b^** | **Fig.2** |
| --- | --- | --- | --- | --- | --- | --- | --- | --- | --- | --- |
| **AMP** | 8.61 | 143.03 | HMDB00045 | 36 | | **Homarine** | 8.73 | 148.50 | Tuffnail *et al.* *^(^*[*^19^*](#_ENREF_19)*^)^* | 26 |
|  | 4.77 | 77.30 |  |  | |  | 8.55 | 149.50 |  |  |
|  | 4.38 | 88.01 |  |  | |  | 8.05 | 129.03 |  |  |
|  | 4.04 | 66.52 |  |  | |  | 7.97 | 130.23 |  |  |
| **ADP** | 4.00 | 65.91 | HMDB01341 | 35 | |  | 4.38 | 49.31 |  |  |
| **ATP** | 4.81 | 77.25 | HMDB00538 | 35 | |  | 3.36 | 36.24 |  |  |
|  | 4.25 | 68.19 |  |  | |  | 2.65 | 58.56 |  |  |
| **ATP/ADP** | 8.58 | 142.90 |  |  | | **Hypotaurine** | 3.36 | 36.24 | HMDB00965 | 16 |
| **AXP** | 8.28 | 155.92 |  |  | |  | 2.65 | 58.56 |  |  |
|  | 6.16 | 89.82 |  |  | | **Isoleucine** | 1.02 | 17.53 | HMDB00172 | 1 |
| **Acetoacetate** | 2.27 | 31.98 | HMDB00060 | 11 | |  | 0.94 | 14.01 |  |  |
| **Alanine** | 3.79 | 53.45 | HMDB00161 | 3 | | **L-Methionine methylsulfonium iodide** | 2.93 | 27.89 | mbse00153  (BMRB) | 20 |
|  | 1.49 | 19.06 |  |  | | **Lactate** | 4.12 | 71.53 | HMDB01311 | 2 |
| **Anserine** | 3.68 | 35.26 | HMDB00194 | | |  | 1.33 | 22.68 |  |  |
|  | 3.19 | 38.52 |  | |  | **Leucine** | 0.97 | 25.03 | HMDB00687 | 1 |
| **Arginine** | 3.25 | 43.43 | HMDB 00517 | | 4 |  | 0.96 | 23.87 |  |  |
|  | 1.92 | 30.31 |  | |  | **Lysine** | 3.03 | 41.90 | HMDB00182 | 5 |
|  | 1.73 | 26.74 |  | |  |  | 1.90 | 32.75 |  |  |
|  | 1.66 | 26.70 |  | |  |  | 1.74 | 29.27 |  |  |
| **Asparagine** | 4.01 | 54.27 | HMDB00168 | | 19 |  | 1.51 | 24.30 |  |  |
|  | 2.87 | 37.51 |  | |  |  | 1.44 | 24.22 |  |  |
|  | 2.94 | 37.59 |  | |  | ***** | 3.76 | 57.33 |  |  |
|  | 2.97 | 37.50 |  | |  | **Maltose** | 3.43 | 72.44 | HMDB00163 | 27 |
|  | 2.85 | 37.41 |  | |  | ***** | 5.42 | 102.80 |  |  |
| **Aspartate** | 3.90 | 55.22 | HMDB00191 | | 17 | **Mannose** | 3.75 | 75.43 | HMDB00169 | |
|  | 2.83 | 39.38 |  | |  | **Methionine** | 2.65 | 31.65 | HMDB00696 | |
|  | 2.80 | 39.48 |  | |  |  | 2.14 | 16.55 |  |  |
|  | 2.69 | 39.41 |  | |  | **Ornithine** | 3.79 | 57.03 | HMDB00214 | 7 |
| **Beta-Alanine** | 3.18 | 39.40 | HMDB0056 | | 15 |  | 3.06 | 41.74 |  |  |
|  | 2.56 | 36.39 |  | |  | **Phenylalanine** | 7.43 | 132.13 | HMDB00159 | 33 |
| **Betaine** | 3.91 | 69.05 | HMDB00043 | | 22 |  | 7.38 | 130.70 |  |  |
|  | 3.27 | 56.35 |  | |  |  | 7.33 | 132.50 |  |  |
| **Choline** | 4.00 | 59.12 | HMDB00097 | | 21 | **Phosphocholine** | 3.23 | 57.06 | HMDB01565 | 21 |
|  | 3.21 | 56.79 |  | |  | **Proline** | 4.14 | 64.08 | HMDB00162 | |
| **Dimethylglycine** | 2.93 | 46.39 | HMDB00092 | | |  | 3.43 | 48.91 |  |  |
| **Glucose** | 5.24 | 95.27 | HMDB00122 | | |  | 3.34 | 49.34 |  |  |
| ***** | 4.65 | 98.99 |  | |  |  | 2.34 | 31.78 |  |  |
| ***** | 3.89 | 63.76 |  | |  |  | 2.06 | 31.88 |  |  |
| ***** | 3.85 | 63.42 |  | |  | **Serine** | 3.99 | 63.18 | HMDB00187 | |
| ***** | 3.71 | 75.36 |  | |  |  | 3.96 | 63.12 |  |  |
| ***** | 3.57 | 74.51 |  | |  |  | 3.85 | 59.32 |  |  |
| ***** | 3.48 | 78.81 |  | |  | **Succinate** | 2.41 | 37.06 | HMDB00254 | 13 |
| ***** | 3.25 | 77.10 |  | |  | **Taurine** | 3.27 | 50.53 | HMDB00251 | 23 |
| **Glutamate** | 2.35 | 36.48 | HMDB00148 | | 12 |  | 3.43 | 38.22 |  |  |
|  | 2.07 | 29.75 |  | |  | **Threonine** | 3.59 | 63.42 | HMDB00167 | 2 |
| **Glutamine** | 2.46 | 33.64 | HMDB00641 | | 14 |  | 1.34 | 22.27 |  |  |
|  | 2.14 | 28.93 |  | |  | **Tyrosine** | 7.20 | 133.90 | HMDB00158 | 31 |
| **Glutathione** | 3.77 | 46.41 | HMDB00125 | | |  | 6.91 | 119.11 |  |  |
| **Glycerophosphocholine** | 4.35 | 62.74 | HMDB00086 | | | **UDP-glucose** | 4.30 | 73.33 | HMDB00286 | |
|  | 3.69 | 69.01 |  | |  | **Valine** | 3.61 | 63.23 | HMDB00883 | 1 |
|  | 3.68 | 65.28 |  | |  |  | 1.05 | 20.79 |  |  |
|  | 3.63 | 65.20 |  | |  |  | 1.00 | 19.50 |  |  |
|  | 3.23 | 57.02 |  | |  | **Unknown A** | 1.10 | 16.93 |  | A |
| **Glycine** | 3.57 | 44.37 | HMDB00123 | | 24 | **Unknown C** | 3.12 | 55.85 |  | C |
| **Glycogen *** | 5.42 | 102.80 | HMDB00757 | | 28 | **Unknown D** | 3.15 | 56.31 |  | D |
| **Histidine** | 7.10 | 119.99 | HMDB00177 | | |  |  |  |  |  |
|  | 4.00 | 57.65 |  | |  |  |  |  |  |  |
|  | 3.25 | 30.73 |  | |  |  |  |  |  |  |
|  | 3.17 | 30.70 |  | |  |  |  |  |  |  |
|  |  |  |  | |  |  |  |  |  |  |

* Resonance with multiple metabolite overlaps

**Supplemental Table 4.** Tables of *p*-values (Student’s t-test, 2-tailed, unequal variance) of PCA scores from the comparison of **A)** LMMB1-G, B, and R (Sup Fig 4), **B)** the south harbor (LMMB1), and the north harbor (LMMB, and LMMB4) of Milwaukee Estuary (Fig 3A, B), and **C)** impacted sites LMMB1, LMMB, LMMB4 and the reference site LMMB5 (Fig 3C). The *p*‑values less than 0.05 are shown in **bold**.

| **A** | LMMB1 | G |  | B |  |
| --- | --- | --- | --- | --- | --- |
|  |  | PC1 | PC2 | PC1 | PC2 |
|  | B | 5.89E-02 | 5.34E-01 | - | - |
|  | R | 2.79E-01 | 9.88E-01 | 4.15E-01 | 5.05E-01 |
|  |  |  |  |  |  |
|  |  |  | LMMB5 |  |  |
| **B** |  | PC1 | PC2 | PC3 |  |
|  | LMMB1 | **9.13E-03** | **1.16E-11** | **5.08E-03** |  |
|  | LMMB4 | **4.62E-02** | 6.16E-01 | 8.35E-02 |  |
|  | LMMB | 4.88E-01 | 8.67E-01 | **1.85E-02** |  |
|  |  |  |  |  |  |
| **C** |  |  | LMMB1 |  |  |
|  |  | PC1 | PC2 | PC3 |  |
|  | LMMB4 | 6.93E-01 | 1.06E-01 | **4.65E-04** |  |
|  | LMMB | 1.37E-01 | 1.09E-02 | **6.50E-03** |  |
